# The Effects of a Functional Food Breakfast on Gluco-Regulation, Cognitive Performance, Mood, and Satiety in Adults

**DOI:** 10.3390/nu12102974

**Published:** 2020-09-29

**Authors:** Sarah J. Kennedy, Lisa Ryan, Miriam E. Clegg

**Affiliations:** 1 Functional Food Centre, Oxford Brookes University, Oxford OX3 0BP, UK; sarah@sometimesoon.co.uk; 2 School of Science and Computing, Galway-Mayo Institute of Technology, H91 T8NW Galway, Ireland; lisa.ryan@gmit.ie; 3 Department of Food and Nutritional Sciences, University of Reading, Reading RG6 6DZ, UK

**Keywords:** functional food, polyphenols, glucose response, insulin response, breakfast, adults, cognition, memory

## Abstract

A whole diet which combines multiple functional foods benefits metabolic risk factors and cognition, but evidence supporting meal to meal benefits, which individuals may find easier to implement, is limited. This study developed a functional food breakfast (FB), using polyphenol-rich ingredients selected for their gluco-regulating and cognitive-enhancing properties, and compared it to a control breakfast (CB). For study 1, total polyphenols were determined using the Folin–Ciocalteu method, and sugar release by in vitro digestion, in frozen and fresh samples. In study 2, healthy adults (*n* = 16) consumed an FB, CB and ready-to-eat breakfast cereal (RTEC) in a randomised crossover design. Glucose (GR) and insulin response (IR), satiety, mood and memory were measured over 180 min. The FB was a rich source of polyphenols (230 mg) compared to the CB (147 mg) (*p* < 0.05), and using frozen muffins did not compromise the polyphenol content or sugar release. Peak GR was highest after the RTEC (*p* < 0.05), and the insulin area under the curve (AUC) was lowest in the FB at 60, 120, 180 min and peak (*p* < 0.05). There were no effects on GR AUC, mood, satiety or memory. Reductions in GR peak and IR following consumption of the FB support the inclusion of functional ingredients at breakfast.

## 1. Introduction

A functional food contains bioactive components which demonstrate health benefits beyond their basic nutritional function [1]. Polyphenols are functional food ingredients found widely in plant foods, including fruits, whole grains and plant extracts [2]. They have been shown to inhibit the activities of key enzymes and glucose transporters in the hydrolysis of starch to glucose [3], and are widely studied for their role in influencing carbohydrate (CHO) digestion and absorption, attenuating postprandial glycaemia and improving insulin sensitivity [4,5,6].

A series of studies by Törrönen et al. [7,8,9] have shown that the addition of berries, rich in polyphenols, to simple sugars significantly lowered the glucose response (GR) and insulin response (IR). Conversely, the addition of berries to starch-rich foods appeared to have little or no effect on GR or satiety [10,11], despite an increase in postprandial antioxidant capacity [11]. Few studies measure insulin, but berries have been found to significantly reduce the IR when consumed with bread [12]. Research around other polyphenol-rich ingredients and their effects on gluco-regulation suggests a role for cinnamon in stimulating insulin activity [13], as well as the potential of baobab to lower the GR and IR [14,15]. The Baobab tree (*Adansonia digitata* L.) is native to Africa but powdered extracts are obtainable from health food shops. The GR- and IR-lowering effects of oat β-glucan are well documented [16], but oats also provide a rich source of polyphenols [17], which could directly contribute to a reduction in starch digestibility [18].

Polyphenols have also been linked to improved cognitive function, notably memory [19,20], related to the ability of flavonoids to cross the blood brain barrier [21,22]. Support from human intervention studies is limited by small samples of mostly at-risk individuals [23,24]. More recent studies in young healthy adults have reported improvements in memory performance following blueberry consumption, suggesting their potential even in individuals with no known risk of cognitive decline [25,26].

Levels of antioxidants in the diet vary depending on factors including growth conditions, food processing and storage [27]. The preparation of polyphenol-rich ingredients may rapidly degrade the polyphenol content [28] or reduce β-glucan solubility, potentially attenuating the blood glucose-lowering benefits [29]. Furthermore, there are few studies investigating the potential synergistic effects on gluco-regulation and cognitive function when multiple functional foods are consumed in practical quantities, as they would be at each meal. Tovar et al. reported the effects of consuming a whole diet based on multiple functional foods, over four [30,31] and eight weeks [32], on cardiometabolic and cognitive outcomes in individuals who were overweight or obese. The ‘active’ diet (AD) used foods selected for their benefits in relation to metabolic parameters and cognition, including blueberries, cinnamon and oats, that were not in the control diet (CD). The authors speculated that improvements in lipid profiles, blood pressure and cardiovascular risk score were attributable to the synergistic actions of the functional ingredients [30]. Furthermore, individuals who consumed the AD performed better on attention and memory tests after a standardised breakfast, compared to the CD [33]. Although promising, there is little evidence to determine whether benefits are present after multiple functional foods are consumed at just one meal, which would be more realistic for individuals to implement. Healthy individuals should be at no immediate risk of metabolic disease or cognitive decline; however, identifying the acute benefits that can be obtained from small dietary changes at breakfast may potentially help them be sustained over time.

This study set out to design a functional breakfast (FB) using ingredients selected for their functional properties, based on quantities that could be realistically consumed at breakfast. To assess the impact of the preparation and storage of the FB on polyphenols and starch degradation, in vitro analysis (study 1) was carried out to compare polyphenol content between the FB and a control breakfast (CB), before and after freezing. The main objective of this research (study 2) was to assess the effect of the FB on GR and IR, satiety, mood and memory in healthy adults when compared with the CB and a commercially available ready-to-eat cereal (RTEC). It was hypothesised that the FB would lower the GR and IR, enhance satiety and mood, and yield more accurate responses on the cognitive tests.

## 2. Materials and Methods

### 2.1. Studies 1 and 2: Overview

Study 1 is the in vitro study which investigated the impact of combining functional food ingredients into a cooked breakfast muffin on polyphenol levels and sugar release. Fresh and frozen samples of the FB and CB were compared to assess the impact of storing the breakfasts in the freezer, in preparation for the in vivo study.

Study 2 is the in vivo study in healthy adults, which assessed the impact of the FB on GR and IR, satiety, mood and cognitive performance compared with a CB and a RTEC.

### 2.2. Studies 1 and 2: Ethics

Study procedures were approved by the Ethical Advisory Committee at Oxford Brookes University, registration number 140836, according to the guidelines laid down in the declaration of Helsinki as revised in 1983. Participants were recruited from advertisements within the university and local press and received monetary compensation for their participation. Written informed consent was received from each participant.

### 2.3. Study 1: Breakfast Development

A CB and FB muffin was developed at the Functional Food Centre (FFC) based on quantities matched to deliver 50 g of available CHO and similar energy and macronutrient contents (Table 1). Functional ingredients selected for their health-promoting properties included oats (antioxidant-rich, GR-lowering), baobab powder (polyphenol-rich, GR/IR-lowering), blueberries (polyphenol-rich, GR/IR-lowering, cognition) and cinnamon (GR-lowering, cognition). Previous in vitro research identified an optimal baobab dose of 1.98 g/50 g available CHO for reducing sugar release in white bread [14]. This amount was also shown to be effective in reducing the IR in vivo [15]. Nutrient profiles were verified by Eurofins Food Testing UK Ltd. and serving sizes were based on these findings. The in vivo study included a third breakfast (Weetos) based on previously collected questionnaire data, which identified it as a commonly consumed breakfast cereal [34].

Dry ingredients were mixed with wet ingredients and measured into greased muffin cases. The CB was baked for 10 min at 200 °C and then a further 8 min at 180 °C. The FB was baked for 10 min at 200 °C and then a further 28 min at 180 °C. The FB serving size (161.8 g) was larger than the CB (140 g) and due to differences between ingredients the FB took longer to cook. Muffins were left to cool for 10 min in the baking tray before transferring to a wire rack for 30 min to cool completely. They were stored overnight in a dark, airtight container and analysed the following morning (fresh samples), or frozen immediately for 24 h and thoroughly defrosted at room temperature before analysis (frozen samples).

### 2.4. Study 1: Total Polyphenols and Sugar Release

Polyphenol analysis of muffin samples was based on a 1 g sample from the frozen CB and FB and the fresh CB and FB. Both samples were extracted with solvent in preparation for polyphenol analysis. For in vitro digestion, muffin samples were weighed out at 2.5 g. All tests were carried out on a minimum of three separate occasions and samples were analysed in triplicate for each test. Total polyphenols were analysed from powdered extract samples and muffin samples using the Folin–Ciocalteu (FCR) method [35]. Polyphenols were extracted from muffin samples.

### 2.5. Study 1: In Vitro Digestion and Sugar Release

Fresh and frozen muffin samples were compared using an in vitro digestion procedure consisting of a simulated gastric digestion phase followed by an ileal digestion phase, with timed sampling at the end of the gastric phase and during the ileal phase [36]. Samples were finely crumbed into 60 mL specimen pots into small uniform pieces (millimetres in diameter) to reflect the chewing process. The pots were inserted into an aluminium heating block and covered with an insulating sheet in readiness for testing. Samples were incubated for 120 min with constant slow mixing, and aliquots taken at 20, 60 and 120 min during ileal digestion. The tubes were centrifuged (1000× *g*, 2 min) in a Biofuge Primo Centrifuge (Heraeus Instruments, Kendro Laboratory Products, Hanau, Germany) and an aliquot of the supernatant was removed for the analysis of reducing sugars. Sugar released from the muffins during digestion was measured by a colorimetric method adapted from Englyst and Hudson [37], designed to measure monosaccharides after an amyloglucosidase secondary digestion to complete the depolymerisation of starch fragments. Absorbance was measured at 530 nm on a Shimadzu UV-1201 spectrophotometer (Shimadzu Corporation, Rydalmere, New South Wales, Australia). Sugar release was measured in mg per g of muffin sample. Previous studies have shown that the in vitro analysis of rapidly digested starch (RDS) can be correlated with the GR in vivo [38].

### 2.6. Study 2: In Vivo Study Design

A three-way repeated measures crossover study design was used with each participant serving as their own control. Randomisation of the order in which the FB, CB or RTEC were consumed was done by the same researcher (SJK) using a random order generator [39]. In the preceding 24 h, unusual vigorous exercise, alcohol, nicotine and caffeine were avoided and after 21:00 h only water was consumed. Test days were on the same day of the week where possible, separated by between two and eight days. All measures were collected within three weeks.

### 2.7. Study 2: Participants

In total, 20 participants were assessed for eligibility. One person was excluded due to allergies and three participants withdrew from the study for personal reasons. Measures were collected from 16 adults. Prior to measurements, participants attended a screening session during which a health questionnaire was completed. Fasting blood glucose, anthropometric measures and blood pressure were measured, confirming that all participants were in good health. Height and weight were measured, and BMI calculated. All measures were collected in the FFC kitchen or laboratory between August 2014 and November 2014.

Exclusion criteria included: (i) diagnosis of medical conditions or medication interfering with metabolism, including diabetes (type 1 or type 2) or neurological illnesses (ii) BMI < 18.5 kg/m^2^ or >30 kg/m^2^, (iii) three consecutive fasting blood glucose readings > 6.1 mmol/L, (iv) post-menopausal, (v) very high polyphenol consumer as identified through food frequency questionnaire [40], and (vi) food intolerance or allergy to breakfast ingredients. 

### 2.8. Study 2: Testing Schedule

**Figure 1:**
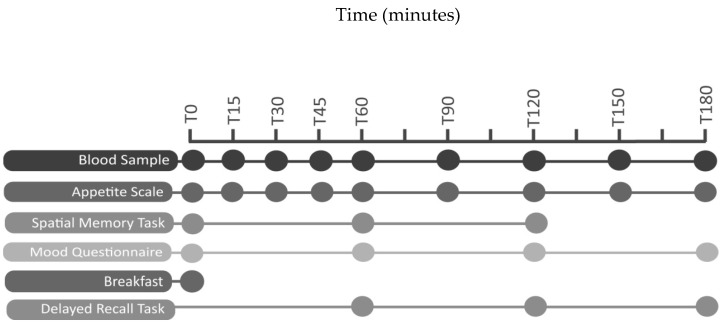
Testing day schedule (Study 2).

The schedule for each testing day was identical (Figure 1). Upon arrival (T0) baseline blood glucose, insulin, appetite, mood and performance on a map task were measured. Further blood measures and appetite scales were taken at 15, 30, 45, 60, 90, 120, 150 and 180 min after the start of breakfast. The map task was completed at 60 and 120 min, and delayed word recall and mood questionnaire were completed at 60, 120 and 180 min. During testing, the previous evening’s dinner was recorded, and participants were asked to replicate this prior to subsequent sessions. A menu reminder was emailed at least 24 h before subsequent test days, and during baseline measures participants recorded their evening meal to check compliance.

### 2.9. Study 2: Test Meals

The CB and FB were prepared by the same researcher (SJK) prior to testing. They were cooled rapidly and frozen for up to five days in a −20 °C freezer, following which they were defrosted overnight in a dark container, to preserve polyphenols, at room temperature. The three breakfasts were coded as one, two or three and participants were blinded until consumption; however, although blinding was maintained, there were clear differences between the ingredients, which made the FB easy to identify. Participants were supervised during eating and finished within 15 min of starting. 200 mL of water was consumed with breakfast and 100 mL was consumed after 90 min.

### 2.10. Study 2: Blood Samples

Glucose and insulin were measured from finger-prick capillary blood samples obtained using a single-use BD Microtainer^®^ contact-activated safety lancet (Bunzl Healthcare, Enfield, UK) at −5 min and 0 (baseline), and post breakfast at 15, 30, 45, 60, 90, 120, 150 and 180 min. 

Blood glucose: After discarding the first drop, 5 μL was drawn into a Hemocue Glucose 201 microcuvette and immediately analysed using HemoCue^®^ 201^+^ analyser (Radiometer Ltd., Crawley, UK). Baseline samples taken in duplicate or triplicate were checked to be within a coefficient of variation of 3%.

Insulin: 300 μL of capillary blood was collected into EDTA-coated microtainers (Bunzl Healthcare, Enfield, UK) and placed immediately on ice. Samples were centrifuged at 4000 rpm for 10 min and 200 μL of the supernatant plasma was removed and placed into 1.5 mL Eppendorphs, then frozen at −40 °C until analysis. Insulin concentrations in the plasma samples were determined by electrochemiluminescence immunoassay using an automated analyser (Cobas^®^ E411; Roche diagnostics, Indianapolis, IN, USA). The Cobas system is a reliable method of plasma insulin determination [41].

### 2.11. Study 2: Cognition, Mood and Appetite

Two cognitive tests were administered: a map task measuring spatial working memory (T0, 60 and 120 min) and a word recall task measuring delayed verbal memory (60, 120 and 180 min).

Spatial working memory systems appear to be predominantly moderated by the hippocampus [42] and have been suggested to be enhanced by polyphenols, particularly in the form of berries, cocoa and isoflavones [19]. Adapted from previous studies in school children and university students [43,44], each map recall task consisted of 30 fictitious countries within five continents. Participants were randomised to nine different categories (e.g., nature at T0, sports at 60 min, and vegetables at 120 min) using a random order generator [39]. Each category used a different map layout to reduce the memory interference observed with a within-participant design. Following pilot work, the difficulty of the task was intensified by increasing the number of words (countries) from 25 to 30 and time to memorise the map was reduced by one minute. The final protocol required each map to be studied for seven minutes on a laptop following which a blank paper map and pencil were provided and two minutes were given to recreate the map from memory.

Verbal memory can be assessed by delayed word recall and has shown sensitivity to interventions assessing glycaemic index (GI), particularly at higher blood glucose concentrations [45]. Participants were asked to write down as many words as they could remember from the previous map task in one minute (maximum score 30). For example, if they had completed the ’nature’ map at baseline then at 60 min they would be asked to recall words, in any order, from the ’nature’ map.

A 20-item mood questionnaire was based on the ‘Activation–Deactivation Check List’ (ADACL) short form [46], which is theoretically derived and reported to have content and construct validity [47]. Items were grouped to measure four components of mood: energy, tiredness, calmness and tension. Participants were asked to rate on a scale of 1 to 4 their subjective feelings of mood at T0, 60, 120 and 180 min (1 = definitely do not feel to 4 = definitely feel). Four basic visual analogue scales (VAS) consisting of a 100 mm line were used to measure participants’ subjective feelings of appetite each time a blood measure was taken [48]. Palatability, namely the pleasantness of the taste and appearance of the breakfast, were recorded once using 100 mm VAS scales 15 min after breakfast.

### 2.12. Study 2: Power Calculation

The method for GR and IR testing used is in line with procedures recommended by the Food and Agricultural Organisation/WHO 1998 and the International Organization for Standardization 26,642 guidelines [49]. These state that to determine the GR of a food, tests should be repeated in a minimum of 10 volunteers. The secondary outcome of the study was cognitive performance. In a previous meta-analysis of the glucose facilitation effect on cognitive performance, a medium overall effect size of *d* = 0.56 was found [50]. Using G*power (Version 3.1.2) for a medium effect size, with α-0.05 (two-tailed) and 0.95 power, a sample size of 16 was required.

### 2.13. Studies 1 and 2: Statistical Analyses

Data were analysed using Microsoft Excel 2010 and SPSS V.22 (Chicago, IL, USA). Values are mean ± SD and significance was set at *p* < 0.05 unless otherwise specified. FCR analyses were compared using one-way ANOVA where pairwise comparisons were performed using Games–Howell adjustment (if the assumption of homogeneity of variances was violated) or Tukey HSD (if the assumption of homogeneity of variances was not violated). In vitro samples were compared using two-way repeated measures ANOVA, where multiple comparisons were performed using a Bonferroni adjustment.

The GR and IR were calculated geometrically as the positive incremental area under the curve (AUC) for each test food, using the trapezoidal rule [49], and included the area above the fasting level only. The normality of the data was tested using the Shapiro–Wilk test. A two-way repeated measures ANOVA (breakfast × time point) was used to compare blood glucose AUC means at 60, 120 and 180 min, and peak differences (defined as the largest change from baseline). Pairwise comparisons were performed using a Bonferroni correction.

Insulin data were analysed using non-parametric Friedman tests based on abnormal distributions.

Map tasks, satiety and mood were analysed using a repeated measures ANCOVA with baseline score entered as a covariate [48]. Map task analyses were performed on the absolute scores for correctly named and located items, items left blank and wrongly answered items. Delayed word recall was analysed using a two-way repeated measures ANOVA (breakfast × time point) on correctly recalled items and blank items. Palatability (taste and appearance) was compared using a one-way repeated measures ANOVA.

## 3. Results

### 3.1. Study 1: In Vitro Study

#### 3.1.1. Total Polyphenol Content of Muffin Samples

FCR analysis of total polyphenols measured in gallic acid equivalents (GAE) showed significant differences between muffin samples (Welch’s F(3,15.7) = 67.53, *p* < 0.001, ŋ^2^ = 0.88). Post hoc analysis using Games–Howell adjustment for multiple comparisons revealed higher total polyphenols in the frozen FB (1.42 mg GAE/g ± 0.10; 229.70 mg ± 15.6) and frozen CB (1.05 mg GAE/g ± 0.02; 147.10 mg ± 3.3) compared to the fresh FB (1.26 mg GAE/g ± 0.06) and fresh CB (1.02 mg GAE/g ± 0.05) (*p* < 0.001). Total polyphenols were also significantly higher in the FB compared to the CB regardless of whether they were fresh or frozen (*p* < 0.001).

#### 3.1.2. Sugar Release 

No significant interactions were observed (time x muffin sample; *p* = 0.255), but there was a main effect between the CB fresh and CB frozen (F(1,5) = 9.25, *p* = 0.029). The CB frozen released more sugar (308.8 ± 174.7 mg/g) than the CB fresh muffin (289.9 ± 161.6 mg/g) (*p* = 0.029). There were no significant interactions or main effects between sugars released from FB fresh or frozen at any phase during the in vitro digestion process (*p* > 0.05).

**Figure 2:**
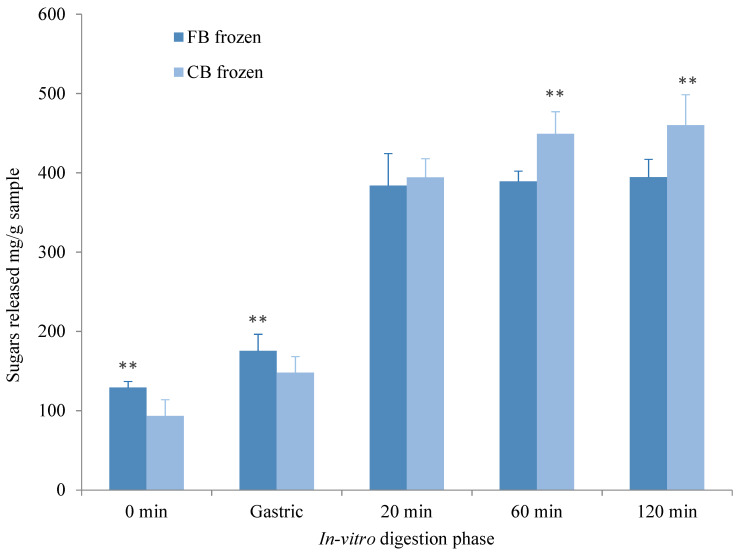
Sugars released between the control breakfast (CB) frozen and the functional breakfast (FB) frozen following in vitro digestion comparison. ** *p* < 0.05.

Comparisons between the CB and FB frozen muffin saw the FB muffin release significantly more sugar than the CB muffin at baseline (F(1,5) = 11.813, *p* = 0.018) and during the gastric phase (F(1,5) = 9.294, *p* = 0.028). Post hoc analyses performed using a Bonferroni adjustment revealed no significant differences during the first 20 min, representing RDS; however, in the intestinal phase of digestion, the FB released less sugars compared to the CB at 60 min (*p* = 0.002) and 120 min (*p* = 0.004), suggesting differences between the amounts of slowly digestible starch (SDS) (Figure 2).

### 3.2. Study 2: In Vivo Study

#### 3.2.1. Participant Baseline Characteristics

The participants were 16 adults (9 males, 7 females). The mean (± SD) age, weight, height and BMI were as follows: 32 ± 10 years, 71.9 ± 8.5 kg, 1.70 ± 0.1 m and 23.8 ± 2.7 kg/m^2^, respectively.

#### 3.2.2. Glucose Response (GR)

**Figure 3:**
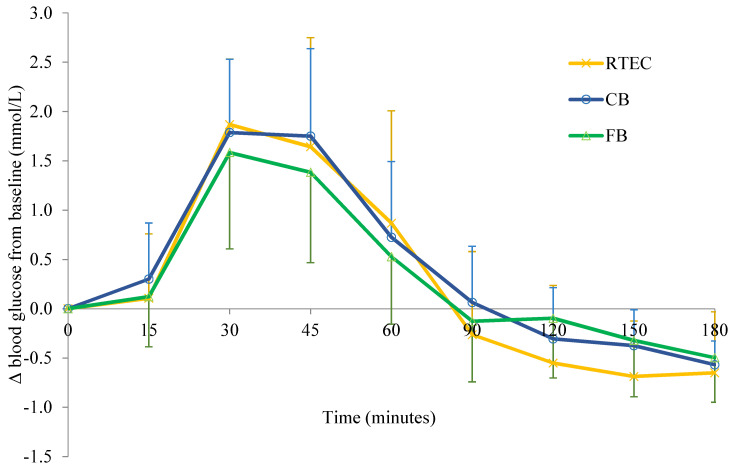
Change from baseline in glucose response (GR) in *n* = 16 adults following consumption of CB (control breakfast), FB (functional breakfast) and RTEC (ready to eat breakfast cereal). Values are mean difference ± SD.

There were no significant differences between GR AUC values at 60 (RTEC 63.34 ± 33.74; CB 63.94 ± 29.02; FB 52.56 ± 33.6 mmol/L/min), 120 (RTEC 84.38 ± 54.95; CB 81.81 ± 42.53; FB 69.08 ± 44.50 mmol/L/min) or 180 min (RTEC 86.63 ± 56.43; CB 83.04 ± 42.66; FB 75.13 ± 51.16 mmol/L/min) (all *p* > 0.05) (Figure 3). However, there was a significant difference between breakfasts in terms of peak glucose concentration (F(2,30) = 4.16, *p* = 0.025). Post hoc analysis using a Bonferroni adjustment revealed a higher peak GR following the consumption of the RTEC (6.55 ± 0.82 mmol/L) compared to the FB (5.99 ± 0.90 mmol/L), with a significant difference of 0.56 (95% CI 0.02 to 1.09) mmol/L (*p* = 0.042).

#### 3.2.3. Insulin response (IR)

**Figure 4:**
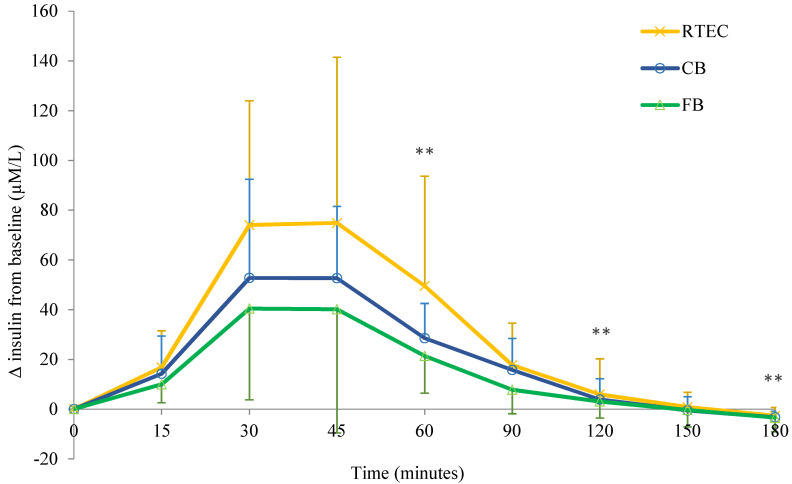
Change from baseline in insulin response (IR) in *n* = 16 adults following consumption of CB (control breakfast), FB (functional breakfast) and RTEC (ready-to-eat cereal). Values are means ± SD. ** *p* < 0.05.

Insulin AUC concentrations were significantly different between breakfasts at 60 (χ^2^(2) = 18.00, *p* < 0.001), 120 (χ^2^(2) = 19.63, *p* <0.001) and 180 min (χ^2^(2) = 19.63, *p* < 0.001) (Figure 4). There were significant differences in absolute insulin concentration (median scores reported) at 60 min between the FB (1034.7 µM/L) and the RTEC (2158.0 µM/L; *p* < 0.001). At 120 min the FB (1329.9 µM/L) was significantly lower than the RTEC (2893.9 µM/L; *p* < 0.001) and the CB (2559.6 µM/L; *p* = 0.040). Again, at 180 min the FB (1457.7 µM/L) was significantly lower than the RTEC (2931.10 µM/L; *p* < 0.001) and the CB (2612.4 µM/L; *p* = 0.040). Additionally, the peak insulin concentration was significantly different between breakfasts (χ^2^(2) = 19.63, *p* < 0.001), where the FB (36.35 µM/L) was significantly lower than the RTEC (75.15 µM/L, *p* < 0.001) and the CB (60.60 µM/L, *p* = 0.040).

#### 3.2.4. Cognitive Tests: Map Task and Delayed Recall

There were no effects of breakfast on cognitive performance for either the map task or the delayed word recall task (Table 2). Furthermore, GR did not significantly correlate with any of the cognitive measures. For delayed word recall there were no significant interactions between breakfast and time for the number of items correctly recalled (F(4,60) = 0.47, *p* = 0.761). Furthermore, breakfast had no effect on the number of items correctly recalled (*p* = 0.784); however, there was a main effect of time, revealing that performance improved at the end of the test. 

#### 3.2.5. Mood, Satiety and Palatability

Mood was not affected by the consumption of any of the breakfasts (*p* > 0.05). There were no significant interactions between breakfast and time in terms of energy (F(4,86) = 0.775, *p* = 0.545), tiredness (F(3,74) = 1.148, *p* = 0.338), calmness (F(3,75) = 0.211, *p* = 0.912) or tension (F(4,86) = 0.523, *p* = 0.719). There was an overall main effect of time on tension (*p* < 0.001), such that tension was scored higher at baseline (9.06 ± 3.55) and then steadily decreased over the testing session by 1.42 points (CI, 0.34 to 2.49, *p* = 0.007) to 7.65 ± 2.84.

Satiety was not affected by the consumption of any of the breakfasts (*p* > 0.05). There were no significant interactions between breakfast and time in terms of hunger (*p* = 0.362), fullness (*p* = 0.662), desire to eat breakfast (*p* = 0.643) or amount of food that could be eaten (*p* = 0.721). As expected, there was a main effect of time on hunger (*p* = 0.015) such that hunger ratings significantly increased over the testing period (*p* < 0.001). There were no significant differences in rating the appearance of the breakfasts (*p* = 0.305); however, there were significant differences in rating taste (F(1.41, 19.75) = 5.34, *p* = 0.022), with a significantly higher rating given to the FB (78.80 ± 14.0) compared to the CB (63.10 ± 18.8, *p* = 0.010) and the RTEC (64.53 ± 19.2, *p* = 0.015).

## 4. Discussion

All three breakfasts were matched to provide 50 g of available CHO, and contained similar levels of energy, fat and protein (Table 1). The FB included functional food ingredients (blueberries, oats, cinnamon, baobab) selected for their potential to improve gluco-regulation [7,8,9,12,13,14,15,16,17,18], as well as ingredients which are advocated as being healthier alternatives (honey, bananas, olive oil) [51]. The combination of these ingredients naturally increased the fibre content of the FB (8.4 g) compared to the CB (2.5 g) and the RTEC (3.7 g). In vitro analysis revealed that the FB released sugar at a slower rate during the later stages of digestion, suggesting there was more SDS in the FB. This could be attributed to the additional fibre, or to the polyphenol levels, which were significantly higher in the FB (229.7 mg) compared to the CB (147.1 mg). SDS energy is released at a slower rate than RDS and therefore should produce a lower GR in vivo [36], which was supported by the in vitro findings but was not seen in vivo. Including in vitro digestion and polyphenol analysis of the CB and FB gives strength to the current study’s findings, but highlights the difficulties in replicating laboratory findings in in vivo studies.

Although polyphenols are easily degraded [28], combining polyphenol-rich ingredients into the FB may have promoted synergism, helping to minimise complete degradation [52]. Furthermore, quickly freezing and thawing the muffins, shown to have a protective effect on β-glucans [29], appeared also to have a protective effect on polyphenols in the FB frozen samples, supporting the interpretation that the freezing procedure in this protocol should not significantly impact GR in vivo.

Consumption of the FB resulted in a significantly lower glucose peak compared with the RTEC, and a significantly lower insulin peak compared with the RTEC and the CB. Additionally, the insulin AUC for the FB was significantly lower than the RTEC at 60 min, and significantly lower than both breakfasts after 120 and 180 min. The addition of polyphenol-rich foods to starch- or sugar-rich CHO meals has been found to improve GR and IR in healthy adults, although to varying extents.

A mixed berry puree sweetened with sucrose improved GR during the early phases of testing and at 150 min, compared with a matched control; however, there was no difference in GR total AUC at 180 min [9]. Polyphenol levels were not measured but the puree was estimated to deliver around 800 mg of polyphenols, much higher than amounts delivered by the FB (230 mg). Furthermore, AUC data at 60 or 120 min were not presented, which would have given further insight into how the response changed over the testing period. There were no glucose-lowering effects of berries added to starch-rich pancakes, and no differences in satiety [10], suggesting that the effects of berries may be attenuated when complex starch compounds are considered, potentially due to the varying extents to which blueberries inhibit starch-degrading enzymes [53]. In three acute studies considering the GR and IR of low-fibre white bread and high-fibre rye bread, the addition of berry puree significantly lowered the IR total AUC in both breads by relatively similar amounts, with a concurrent early phase reduction in GR AUC [12]. The amount of berries used (150 g) was considerably higher than in the current study (30 g), and although the polyphenol content was not reported, findings suggest that the addition of berries may improve gluco-regulation, in addition to the GR and IR benefits of the consumption of a high-fibre, low-GI meal. The addition of baobab into white bread reduced the IR AUC over 180 min and insulin peak, compared to a green tea-enriched and a control bread [15]. The baobab extract used was the same as in the current study, and delivered 61 mg of added polyphenols, although total polyphenol content after baking was not measured. It appears there is a role for baobab in facilitating a lower postprandial IR, linked to high levels of tannins, flavonoids and soluble fibre, but differences in GR have so far only been reported in vivo and in much higher quantities (18.5 g/50 g available CHO) than were used in the FB [14].

A review concluded that there is overall support for the addition of polyphenol-rich sources in order to reduce peak IR and sustain IR, particularly when added to bread [4], so the combination of ingredients in the FB may have helped to maintain a lower IR over the whole testing period. This is encouraging as it supports promoting the consumption of a range of functional foods in realistic amounts, rather than consuming large, often unrealistic amounts of one food.

Törrönen et al. [9] reported that the berry puree significantly reduced peak glucose by 1.0 mmol/L, which they suggested could be of clinical significance. In the current study, a smaller reduction in peak glucose was observed between the FB and CB (0.53 mmol/L), which was approaching significance (*p* = 0.099), suggesting a potential dose response of polyphenols to reduce peak glucose. A reduction in peak glucose may be more important than lowering overall GR for the development of cardiovascular complications [54], which, based on current findings, has implications for deciding the composition of breakfast to consume.

There were no significant differences between scores for either the map task or the delayed recall task, regardless of which breakfast had been consumed. A strength of this study was the collection of blood measures to attempt to explain mechanisms; however, there were no significant associations between cognitive performance and GR or IR. To date, only one study has considered the effect of multiple functional ingredients on cognitive performance, wherein improvements in memory were observed in adults following the consumption of a multi-functional diet (AD) compared to a CD [30]. Benefits were partly attributed to the inclusion of blueberries, which were provided in greater quantities (75–95 g) than in the current study (30 g). Cinnamon was also included, but at higher quantities (3 g) than in the current study (1 g). The polyphenol content was not measured, but the AD was consumed over four weeks, suggesting that measurable improvements may be associated with the regular consumption of functional foods.

Improvements in some aspects of memory were observed in adults with myocardial infarction following the consumption of blueberry juice [24]; however, over 1000 mg of polyphenols were consumed per day, suggesting that much larger doses, potentially deliverable from juices, may be needed to observe an effect. Healthy adults consuming blackcurrant extracts, containing 525 mg polyphenols per 60 kg bodyweight, performed better on attention tasks compared to a sugar-matched control [26]. However, this was specific to whether the juiced or powdered extract was consumed, suggesting that the way an extract is prepared can influence physiological and cognitive outcomes. The current study provided polyphenols via the whole fruit, ground flour and a powdered extract; therefore, it is unclear what influence this may have had, if any, on cognitive performance.

The cognitive tests used in this study were carefully selected [42] based on cognitive domains that have shown sensitivity to the effects of an oat-based breakfast compared to no breakfast [43], and on reliability as derived from their use in previous studies [43,44]. The map task was extended from the original version [43] to increase the difficulty relative to adults, and to account for the increased frequency of delivery. When used previously with university students to compare the effects of a snack [44], an additional test ran concurrently, thus increasing the task complexity. If future studies consider using the map task, this could form part of study designs. Map scores indicated a trend for performance to increase over time, suggesting a potential practice effect, despite the use of different map layouts and the random order allocation of the tasks, which was expected to have accounted for some of this. As part of the development process, the cognitive tests were successfully piloted in a sample university population; however, during the study a ceiling effect was observed whereby three participants obtained maximum marks on some map tasks, subsequently reducing the overall power of the study’s cognitive findings. This may have been due to differences in the age of acquisition of verbal material, the educational attainment between participants [55], or in implicit learning strategies, which were not measured as part of the study.

Soluble fibre, particularly β-glucans, increase viscosity, and their solubility may promote satiety [56]. Breakfasts varying in β-glucan (2.2 g to 5.7 g) seem to cause a significant decrease in IR and an increase in satiety, in a dose-responsive manner [57]. These values were higher than the amount of β-glucan provided by oats in the FB (estimated to be at least 1.5 g), and despite an additional contribution from baobab, the values were perhaps still too low to have an appreciable effect on satiety.

The effect of food on mood is strong, and it is well established that alleviating hunger through eating releases endorphins, which improves mood [47]. In the current study there was a decrease in reported feelings of tension following the consumption of either breakfast, which continued over the testing period. Mood can be influenced by GR, and higher blood glucose levels have been associated with feeling less tense [58]. Breakfasts higher in whole grains and fibre have been shown to positively influence mood compared to refined breakfasts [59]; however, similar to the current study, not all research has observed an effect [60,61,62].

Palatable food releases endorphins which may induce a positive mood and which can interact with satiety, potentially increasing cognitive efficiency [58], and although the FB was rated as tasting significantly more pleasant than the CB or the RTEC breakfast, this appeared to have no effect on performance or ratings.

All breakfasts were matched for available CHO, energy, fat and protein; therefore, significant differences could be attributed to the inclusion of the polyphenol-rich ingredients, although this research did not set out to identify which ingredient specifically. Furthermore, as comparable studies measure total polyphenols, no in-depth characterisations of polyphenols were completed, so findings should be interpreted with these limitations in mind. Finally, habitual breakfast frequency was not measured, which may have potentially affected performance on cognitive tasks [63].

## 5. Conclusions

There is widespread support for the benefits of consuming functional ingredients, but in amounts that are generally unrealistic in a single meal. The current study adds to the literature by reporting the acute benefits to gluco-regulation that can be obtained from the inclusion of functional food ingredients at breakfast, in healthy (normal-weight) adults. Promoting the consumption of polyphenol-rich foods as part of a healthy breakfast could be considered. The effects of a single meal over a sustained period warrant further investigation.

## Figures and Tables

**Table 1:** Nutrients and ingredients of the breakfast meals (per serving).

	Unit	RTEC ^a^	CB ^b^	FB ^b^
Serving size	g	60.0	140.0	161.8
Serving size (milk)	mL	180.5		
Nutrients ^c^				
Energy	kJ	1475	1456	1406
	kcal	353	344	335
Protein	g	10.8	11.3	11.2
Fat	g	9.4	10.5	8.1
Total carbohydrate	g	53.7	52.5	58.4
Total sugars	g	22.6	10.6	14.1
Total fibre (AOAC)	g	3.7	2.5	8.4
Available carbohydrate	g	50	50	50
Beta-glucan from oats	g			1.9
Ingredients				
Weetabix weetos	g	60	-	-
Scott’s porridge oats	g	-	-	53
Dr.Oetker baking powder	g	-	5	5
Tesco olive oil	g	-	-	2.6
Tesco British whole milk	mL	180.5	50.0	40.0
Tesco everyday value bananas	g	-	-	30
Schwartz ground cinnamon	g	-	-	1.0
Tesco blueberries	g	-	-	30
Minvita baobab superfruit powder	g	-	-	1.98
Rowse clear honey	g	-	-	11
Filippo Berio light olive oil spray	g	-	0.7	0.7
Tesco free range eggs (whites)	g	-	32	30
Allison Strong White Bread Flour	g	-	57	-
Tesco English unsalted butter	g	-	8.4	-
Tate & Lyle granulated white sugar	g	-	3.0	-
Myprotein fructose powder	g	-	6.81	-

^a^ Nutrients as per manufacturer labels, ^b^ Nutrient profiles obtained from Eurofins Food Testing UK Ltd., ^c^ Values for RTEC include milk. RTEC, Ready to Eat Cereal; CB, Control Breakfast; FB, Functional breakfast; AOAC, Association of Analytical Chemists.

**Table 2:** Mean scores ± SD for map task (T0, T60 and T120; top table) and delayed recall task (T60, T120 and T180; bottom table) following consumption of RTEC, CB and FB.

**Map**	**T0**			**T60**			**T120**			
Items	RTEC	CB	FB	RTEC	CB	FB	RTEC	CB	FB	*p*-value
Correct	18 ± 8	19 ± 8	17 ± 8	20 ± 7	20 ± 8	18 ± 8	20 ± 8	20 ± 9	19 ± 8	>0.05
Wrong	2 ± 2	2 ± 2	3 ± 2	2 ± 2	2 ± 2	2 ± 2	2 ± 2	2 ± 2	2 ± 2	>0.05
Blank	10 ± 6	8 ± 6	10 ± 7	9 ± 6	7 ± 6	10 ± 6	8 ± 6	8 ± 7	9 ± 6	>0.05
**Recall**	**T60**			**T120**			**T180**			
	RTEC	CB	FB	RTEC	CB	FB	RTEC	CB	FB	*p*-value
Correct	13 ± 3	13 ± 5	13 ± 5	12 ± 5	13 ± 5	12 ± 6	16 ± 5	15 ± 5	15 ± 4	>0.05
Wrong	0 ± 0	0 ± 1	0 ± 1	0 ± 1	0 ± 0	0 ± 1	0 ± 0	0 ± 1	0 ± 1	>0.05
Blank	17 ± 4	16 ± 6	18 ± 5	18 ± 4	15 ± 6	18 ± 6	14 ± 5	14 ± 6	15 ± 4	>0.05

Abbreviations: RTEC, ready to eat breakfast cereal; CB, control breakfast; FB, functional food breakfast.
